# The importance of central sensitization for clinical trials of disease modifying osteoarthritis drugs (DMOADs)

**DOI:** 10.1016/j.ostima.2025.100261

**Published:** 2025-02-18

**Authors:** David A Walsh, Daniel F McWilliams

**Affiliations:** a Pain Centre Versus Arthritis & Academic Rheumatology, University of Nottingham, UK; b Advanced Pain Discovery Platform, UKRI, London, UK; c NIHR Nottingham Biomedical Research Centre, Nottingham, UK

**Keywords:** Pain, Nociplastic, Central sensitization

## Abstract

Osteoarthritis (OA) pain is associated with structural changes in the joint, which are usually quantified by imaging techniques. It is anticipated that structural disease modifying OA drugs (DMOADs) would reduce the burden of OA pain. However, nociceptive pain is moderated by the central nervous system. Central sensitization, increased activity in central nervous system neurones in response to a standard nociceptive input, is one reason why disease modification might not effectively relieve OA pain. Central sensitization may result from facilitated central neuronal activity, or inadequate inhibition by endogenous analgesic mechanisms. It changes the experience of pain: its severity, distribution and qualities, and its emotional and cognitive dimensions. Central sensitization can be a barrier to analgesic benefit from treatments directed at joint structure, and central pain processing can obscure analgesic benefit from structural modification in randomised controlled trials. Indices of central pain hypersensitivity might reflect central sensitization in humans. They include self-report questionnaires such as the Central Aspects of Pain (CAP) and short form Central Sensitization Inventory (CSI-9), and quantitative sensory testing (QST) modalities of Pressure Pain detection Thresholds distant to the affected joint, Temporal Summation, and Conditioned Pain Modulation. Understanding, measuring, managing and adjusting for central pain hypersensitivity should increase the power of clinical trials to demonstrate that DMOADs not only improve joint imaging outcomes, but also improve pain, the predominant clinical problem of OA.

## Why are structural disease modifying osteoarthritis drug (DMOAD) clinical trials necessary?

Osteoarthritis (OA) remains a major burden: for the individuals who suffer from it, for healthcare providers and for society. Pain is the predominant symptom of OA, and OA pain is driven by biomechanical, structural, cellular and biochemical factors. Joint replacement surgery is one of the most effective interventions for any condition, but is not universally successful [1]. Non-surgical approaches to managing OA remain essential. Analgesics often provide inadequate benefit for OA pain and may themselves cause serious adverse events.

Medical treatments that can slow or reverse the structural pathology of OA have great potential to also reverse or slow pain progression. While our understanding of OA joint pathology has increased substantially over recent years, interventions that might retard OA structural progression have not produced convincing symptomatic benefit [2]. This is likely, at least in part, to be due to central pain mechanisms. Seeing what a joint looks like on imaging is only of value if that knowledge can enable symptomatic improvement. FDA approval of DMOADs therefore requires that they also improve symptoms.

## Why is pain improvement not an inevitable consequence of reducing joint pathology?

Several explanations might contribute to an apparent disconnect between structural disease severity and symptoms. Not all structural changes cause pain. Some might reflect adaptive reparative responses. Osteophytes represent new bone that forms around the margins of osteoarthritic joints. Osteophytes can be a source of pain, but they might only rarely be a major driver of symptoms [3]. Pain is strongly influenced by contextual factors, such that measured benefits from placebos often approximate what might be expected from DMOADs or pharmaceutical analgesics [4]. People will often decide to enter clinical trials when pain is at its worst, and the fluctuating nature of OA pain tends towards improvement with regression to the mean.

Another key factor results from moderation by the central nervous system (CNS) of nociceptive signals from the joint. CNS processing in people with OA can augment nociceptive signalling or impair the body's ability to suppress pain. For some people, central pain processing may have a larger impact on their pain than does joint pathology. Adverse central pain processing can be a barrier against symptomatic response to DMOADs and may obscure the benefits that DMOADs could offer. Central pain hypersensitivity might persist even when joint pathology has resolved [5].

## CNS pain mechanisms in OA

Processing of nociceptive signals occurs at multiple levels within the CNS, all of which may be potential targets for treatment. Increased response of CNS neurones to a standard nociceptive input specifically defines central sensitisation [6], and altered brain processing also contributes to the affective and cognitive dimensions of OA pain. Central sensitization can be measured directly in animal models by electrophysiology. Increased neuronal responsiveness has been investigated most extensively in first order neurons in the dorsal horn of the spinal cord but may also occur at supraspinal levels. Descending signals associated with thalamic and brainstem activity can either augment or suppress spinal nociceptive neurones. Descending facilitation and impaired inhibition both contribute to central sensitization. Central sensitization is not a single entity and cannot be captured by any single measurement tool. Its complexity reflects the complexity of pain itself.

In humans, only indirect measurement of central sensitization is ethically acceptable, although measures of `central pain hypersensitivity’ may be indicative of central sensitization. Quantitative sensory testing (QST) may measure evidence of pain hypersensitivity in response to a standardised nociceptive or non-nociceptive stimulus [7] . Self-report questionnaires can capture symptoms or comorbidities linked to central pain processing [8,9].

Central sensitization is one of the mechanisms that might contribute to ‘nociplastic pain’. Nociplastic pain is pain that arises from altered nociception despite no clear evidence of actual or threatened tissue damage causing the activation of peripheral nociceptors or evidence for disease or lesion of the somatosensory system causing the pain [6]. Questionnaires and QST might together permit evaluation against criteria for nociplastic pain [10]. However, nociplasticity, like central sensitization, occurs over a spectrum. Thresholds for dichotomous classification or `diagnosis’ of nociplastic pain are currently under investigation, but await full validation.

Pain is both a sensory and an emotional experience. What humans recognise as pain depends on interconnections between several brain regions, rather than activity in any single brain nucleus [11]. Altered brain functional connectivity revealed by functional Magnetic Resonance Imaging may contribute to the emotional components of chronic OA pain.

Chronic OA pain is associated with sleep disturbance, diurnal fatigue, impaired or unhelpful cognitions, anxiety and low mood. These central aspects of pain may reflect a generalised CNS dysfunction [9], and are associated with pain spreading beyond the site of OA pathology. They are linked with pain qualities such as burning or shooting, and with pain in response to non-damaging stimuli (allodynia). Similar pain qualities are found in neuropathy (nerve damage). They might indicate nociplastic pain when they are not adequately explained by tissue pathology or nerve damage, for example in fibromyalgia [10].

## Evaluating central pain hypersensitivity in OA

Central pain hypersensitivity may be evaluated by combining static and dynamic QST modalities [7]. Pressure Pain detection Threshold (PPT) at the knee is a static QST modality that might reflect overall sensitivity from a combination of peripheral sensitization in joint tissues and altered CNS nociceptive processing. PPT distant from an affected joint might better reflect central pain hypersensitivity [7]. Dynamic QST modalities measure changing pain in response to repetitive or continuous nociceptive stimulation [7]. Temporal Summation (TS) describes an increased pain sensitivity with repeated application of a standard nociceptive stimulus (e.g. punctate stimulation of the skin). Conditioned Pain Modulation (CPM) records changing pain sensitivity (e.g. measured by PPT) when a concurrent painful stimulus is applied to another body site, for example by cold water emersion or by induced ischaemia [7]. PPT is usually increased (less sensitive) under the influence of a heterotopic nociceptive stimulus, but a reduction or even reversal of this CPM response might reflect deficient endogenous analgesic control.

The Central Aspects of Pain questionnaire (CAP, [9]) records 8 characteristics that have been associated with QST evidence of central pain hypersensitivity in people with knee OA. It measures a single factor (CAPf) that is associated with OA pain severity and predicts poor prognosis in people with knee pain. CAPf might indicate a global CNS dysfunction associated with central sensitization, but might also indicate psychological and physiological consequences of chronic OA pain. The 9-item short form of the Central Sensitization Inventory (CSI-9) was developed to measure symptoms and comorbidities believed to be associated with central sensitization. Its items converge with those in CAP, and it too has been associated with QST evidence of central pain hypersensitivity in people with knee OA [8].

Both intermittent and constant OA pain are high in people with high indices of central pain hypersensitivity [12], although intermittent pain might be more specifically driven by peripheral pathology or physical activity. Evidence of central pain hypersensitivity may be most prominent in people with longstanding OA and severe pain. It remains unclear to what extent these associations represent causal effects of sensitization on OA pain, or consequences of chronic pain. QST evidence of central pain hypersensitivity may reverse on successful removal of peripheral nociceptive input, for example by joint replacement surgery [13]. However, those for whom pain persists often continue to display worse scores on questionnaires that address central pain hypersensitivity [13].

Different QST modalities are designed to assess different components of central sensitization, and therefore statistical associations between indices of central pain hypersensitivity are often weak, or non-significant. Static QST modalities might be most strongly associated with current pain, whereas dynamic QST modalities might better predict OA pain prognosis [7]. Static and dynamic QST modalities might identify discrete subpopulations of people with OA with different underlying central pain mechanisms [14]. Measurement tools therefore must be carefully selected and matched to the purpose and population to which they are applied.

## Joint pathology drives central sensitization

**Fig. 1:**
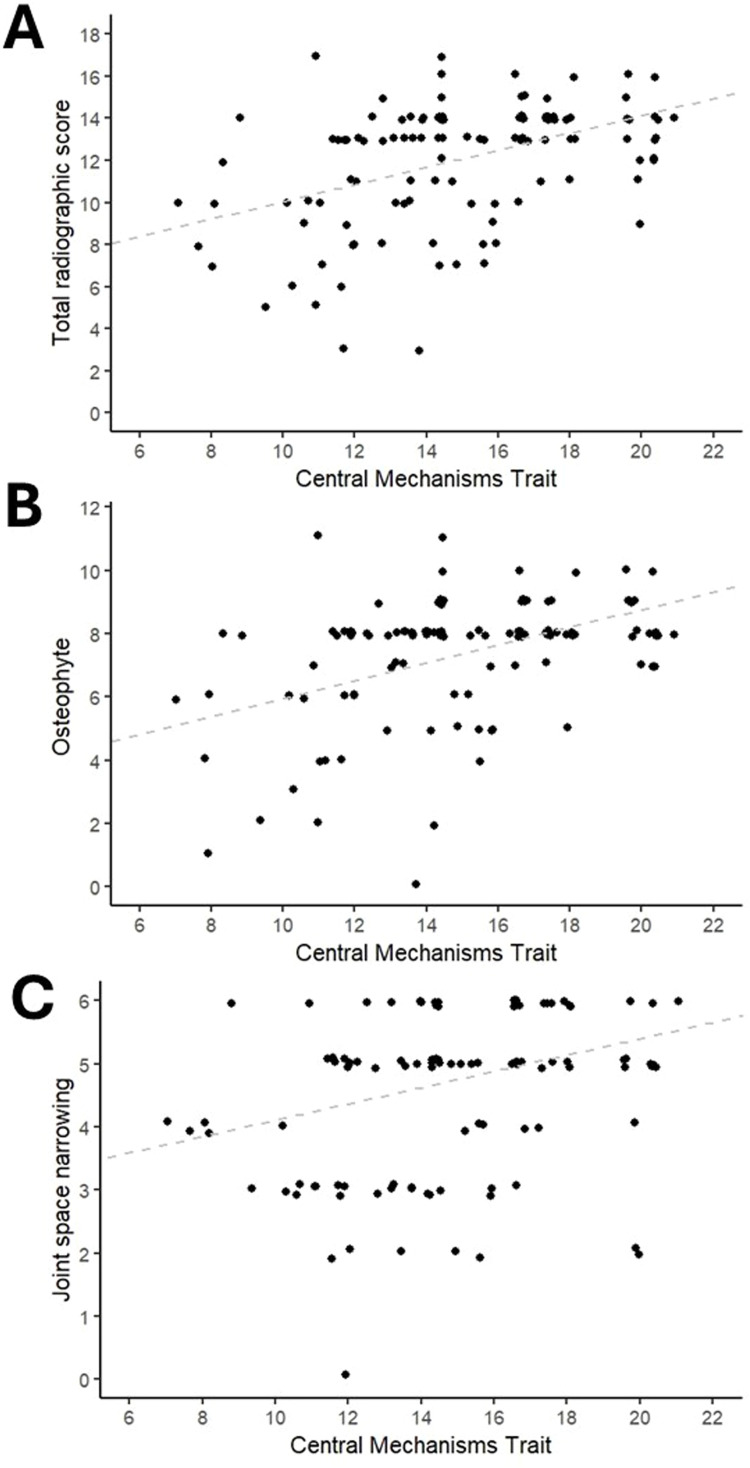
Radiographic OA structural severity is associated with symptoms of central pain hypersensitivity in people with knee OA. Scatterplots of Central Mechanisms Trait (CMT, 8-item, self-reported score validated for measuring central pain hypersensitivity in people with knee pain [9]) against radiographic OA structural severity (calculated by the method of Nagoasa [20]). **A**. CMT is higher in people with more severe radiographic total score (Spearmans rho = 0.45, *p* < 0.001). **B**. CMT is higher in people with more severe osteophyte (OST) score (Spearmans rho = 0.42, *p* < 0.001). **C**. CMT is higher in people with more severe joint space narrowing (JSN) score (Spearman's rho = 0.34, *p* < 0.001). Data taken from the Sherwood Forest/University of Nottingham Human Bone and Joint Tissue Repository (for additional details see: https://www.nottingham.ac.uk/paincentre/resources/joint-tissue-repository.aspx). All participants were undergoing pre-operative assessment for knee OA (*n* = 126). Radiographic scores calculated by a single observer from a standardised, semi-flexed, anteroposterior image. Possible ranges of the scores were CMT: 0 to 24 (6 to 22 presented), JSN: 0 to 6, OST: 0 to 12, radiographic score 0 to 18 (JSN+OST). Lines of best fit added to the plots.

Central sensitization may be driven by chronic nociceptive input which, in turn, depends on joint pathology and mechanical stimulation. Not all imaging markers of joint pathology have been associated with central pain hypersensitivity, and, conversely, some apparent associations might be explained by concurrent pathology or confounders. The evidence base for this is currently limited, although some studies have yielded important hypotheses. Radiographic joint space structural changes and evidence of synovitis, have been associated with evidence of central pain hypersensitivity (Fig. 1), although bone marrow lesions were not [15]. These apparent differences might reflect different contributions of various joint structures to chronic nociceptive input. For example, bone marrow lesions might be specifically associated with intermittent, weight bearing rather than constant OA pain [16]. Joint pathology might be an important, but not exclusive, driver of central sensitization, which might also be influenced by genetic constitution, sex, comorbidities, aerobic fitness and antidepressant medications.

## Improving clinical trial design for DMOADs by considering central sensitization

Incorporating an understanding of central pain processing could improve the ability of randomised controlled trials to demonstrate clinical benefit from DMOADs. DMOADs might be expected to improve pain that is linked to joint-related factors, such as weightbearing pain that is driven by OA structural pathology, but less likely to improve those driven by central pain mechanisms. Primary outcome measures in clinical trials should focus on predominant concerns of those patients for whom the new treatment is intended. Modification of OA structural changes might not be sufficient to judge a trial as a success if not accompanied by symptomatic benefit. DMOAD trials might therefore target recruitment at people with predominantly nociceptive pain and little evidence of central sensitization.

DMOADs might have greatest potential for analgesic benefit in people with early OA. A higher proportion of pain might be attributed to structural pathology in the early stages of the disease. Evidence of central pain hypersensitivity tends to be less in people with early than those with longstanding OA [17]. Early OA is dominated by intermittent pain, often occurring during activity. Weightbearing pain is associated with subchondral bone marrow lesions [16]. DMOAD trials, including those targeting BMLs, might most efficiently recruit people with early OA when seeking to improve pain.

In end-stage structural OA, central sensitization might be a barrier to analgesic response to peripherally targeted interventions, for example joint replacement surgery [1]. Recruiting people with low indices of central pain hypersensitivity might increase efficiency of DMOAD trials in late OA. Optimising central pain processing prior to participation in DMOAD trials might be ideal, but relieving central sensitization while peripheral nociceptive drive persists might not be feasible. Targeted recruitment to clinical trials, through comprehensive pain phenotyping, might increase their power but reduce the generalisability of their findings to broader OA populations in clinical practice.

Placebo response may explain the majority of patient benefit from current OA treatments [18]. Placebos augment descending inhibition of nociceptive transmission [19]. Clinicians should embrace opportunities to augment endogenous analgesic pathways which have the potential to synergise with effective DMOAD treatment. However, large and variable placebo responses can conceal any additional benefit from DMOADs in clinical trials. Factors other than placebo effects also influence central pain processing, and also change over the several years required to demonstrate structural disease modification. Central pain hypersensitivity is therefore a variable that might be measured, tracked and adjusted for when seeking to demonstrate effects of DMOADs on OA pain. However, reducing nociception can itself reduce central sensitization, and over-adjustment might further obscure real benefit from DMOAD treatment. Even if not used for statistical adjustment, central pain hypersensitivity indices (such as QST and self-report instruments) will often give important context to help interpret trial findings and assist in the design of future studies.

## Conclusions

Central sensitization modifies the OA pain experience. For some, central pain hypersensitivity might be the major driver for OA pain and can explain persistent pain despite effective management of OA joint pathology. It can be a barrier to symptomatic benefit from DMOADs in clinical trials. Central pain hypersensitivity is a dynamic state, rather than a fixed trait, and adds noise that can obscure clinically important pain relief. Central pain hypersensitivity is dependent on continuing nociceptive drive, and is also modified by lifestyle and other factors that cannot remain fixed during lengthy DMOAD clinical trials. Understanding the importance of central sensitization to the OA pain experience can inform targeted participant recruitment, pain assessment, and adjustment for confounding. Measuring and addressing central pain hypersensitivity should be a key component of all DMOAD trials.

## Declaration of competing interest

**DFM –** Grant support from Orion Pharma, Eli Lilly & Company, Pfizer

**DAW –** Grant support from Pfizer Ltd, UCB, Orion Pharma, GSK, Eli Lilly. Grant support from non-pharma sources of Versus Arthritis, Nuffield Foundation, Thalidomide Trust, UKRI, NIHR. Consulting fees from Gruenenthal, Contura International and AbbVie. Honoraria from Pfizer and Medscape international. Director of UKRI/Versus Arthritis Advanced Pain Discovery Platform.
